# Clinical Performance of the Automated LIAISON® Meridian *H. pylori* SA Stool Antigen Test

**DOI:** 10.1155/2020/7189519

**Published:** 2020-03-19

**Authors:** Antone R. Opekun, Claudia Zierold, Ashli Rode, Frank A. Blocki, Giulia Fiorini, Ilaria Maria Saracino, Dino Vaira, Fred M. Sutton

**Affiliations:** ^1^Division of Gastroenterology and Hepatology, Department of Internal Medicine, Baylor College of Medicine, Houston, TX 77030, USA; ^2^Division of Gastroenterology, Nutrition and Hepatology, Department of Pediatrics, Baylor College of Medicine, Houston, TX 77030, USA; ^3^DiaSorin Inc., 1951 Northwestern Avenue, Stillwater, MN 55082, USA; ^4^Department of Medical and Surgical Sciences, “S. Orsola” Hospital, University of Bologna, Bologna, Italy

## Abstract

**Background:**

Antigens derived from *Helicobacter pylori* can be used as stool biomarkers to assist in the diagnosis of *H. pylori* infection. Since current assays have variable performance, we assessed the clinical performance of the automated LIAISON® Meridian *H. pylori* infection. Since current assays have variable performance, we assessed the clinical performance of the automated LIAISON® Meridian

**Methods:**

This prospective multisite study enrolled patients undergoing an esophagogastroduodenoscopy with collection of biopsy and stool specimens. Adult patients (≥22 years) participated in the study from February 2017 to August 2018. Specimens of the stomach were tested by three methods, known as the Composite Reference Method: (1) histological evaluation, (2) culture of the organism, and (3) rapid urease detection test. *H. pylori* infection. Since current assays have variable performance, we assessed the clinical performance of the automated LIAISON® Meridian *H. pylori* infection. Since current assays have variable performance, we assessed the clinical performance of the automated LIAISON® Meridian

**Results:**

277 patients (63% female) were included in the study. The prevalence of infected subjects was 24.2% in this study cohort. Clinical performance assessed against the Composite Reference Method showed very good agreement (Cohen′s kappa = 0.922), with good sensitivity (95.5%) and specificity (97.6%). Reproducibility study results showed total imprecision ranging from 3.1% to 13.9% CV.

**Conclusion:**

The automated LIAISON® Meridian *H. pylori* SA assay brings reliable noninvasive testing for *H. pylori* to the laboratory that is in very good agreement with the current, more invasive biopsy-based methods such as histology, culture, or rapid urease test. The clinical trial identifiers are NCT03060746 (pretherapy) and NCT03060733 (posttherapy).*H. pylori* infection. Since current assays have variable performance, we assessed the clinical performance of the automated LIAISON® Meridian *H. pylori* infection. Since current assays have variable performance, we assessed the clinical performance of the automated LIAISON® Meridian

## 1. Introduction

The accurate diagnosis of *Helicobacter pylori* (*H. pylori*) remains clinically important due to its association with several gastroduodenal diseases including peptic (duodenal and gastric) ulcer disease, gastric lymphoma, and gastric cancer [1]. For much of the 19^th^ and 20^th^ centuries, peptic ulceration was thought to be related to stress and excessive production of stomach acid [2]. Following the discovery of *H. pylori* as a causal agent of peptic ulcers, poor hygiene, crowded conditions, sharing contaminated water supplies, and interfamilial fecal-oral transmission were determined to be important factors in the unwitting transmission of the condition [3]. Although the incidence of gastroduodenal diseases, including gastric cancer, appears to have declined in areas where hygiene has sufficiently improved to limit *H. pylori* transmission [4], the prevalence of *H. pylori* infection and reinfection continues to be a worldwide problem [5], and accurate, noninvasive, and convenient diagnostics are needed.

The Japanese healthcare system recently approved the insurance coverage of *H. pylori* infection diagnosis and eradication in all patients [6]. Such testing has not yet been established as an official guideline in the U.S., even though more than twenty thousand patients are diagnosed annually with gastric cancer and approximately half are expected to succumb to it [7]. Effective screening for active and occult infection is essential in the diagnostic algorithm, not only for the treatment of symptomatic disease but for the prevention of future malignancies. However, the ideal screening test approach has not yet been achieved and widespread reduction in disease by identification and elimination of *H. pylori* as a pathogen remains paramount [8].

A number of tests for *H. pylori* infection are currently available, each with their own advantages and disadvantages. Serological testing, while being noninvasive and relatively simple to perform, is frequently vulnerable to poor specificity and sensitivity and unable to accurately discriminate between active and past infections [9]. Endoscopy with biopsy collection for the assessment of *H. pylori* infection through histology, rapid urease test and culture, or point-of-care urease breath testing is considered to be the gold standard in the diagnostic algorithm [10], but such invasive testing is expensive, time-consuming, and not readily available to those most at risk. Furthermore, endoscopic screening carries unacceptable risks when used without specific clinical indications. Isotopic tracer-labeled urea breath testing that exploits the high bacteria-associated urease activity associated with active *H. pylori* infection was developed to complement endoscopic sampling and is often used to assess eradication treatment efficacy [11]. The ^13^C or ^14^C urea breath test is safe and noninvasive, but it requires either point-of-care testing with desktop instruments and qualified personnel or shipment of collected samples to an analytical laboratory [12]. This is often considered too costly to implement for widespread screening. Furthermore, the test is highly influenced by concomitant or recent antibiotic or acid-blocking proton pump inhibitor (PPI) medication uses [13]. These medications suppress infectious activity and associated intragastric urease expression and thereby diminish test sensitivity. Stool antigen tests (SATs) are also noninvasive diagnostic tools and offer advantages [14]; most importantly, they were thought to be less likely impacted by the recent use of antibiotics or H2-antagonist acid blockers when used as a bridge from PPI therapy [15].

The first approved and commercially marketed SATs were introduced after serum serological tests were developed [16, 17]. The early tests used polyclonal antibodies as reagents and were hampered by false-positive results, especially in the setting of posttreatment assessments [18, 19]. Furthermore, polyclonal tests were found to be problematic and inaccurate in children [20, 21]. Subsequently, reagents were refined and monoclonal antibody-based techniques were found to have higher specificity [22, 23]. Sensitivity and specificity of monoclonal SATs have been exceeding 80% [24], but this is considered suboptimal since approximately 20 percent of patients testing falsely positive could be inappropriately exposed to treatment that is expensive and has side effects. This scenario might evoke the use of a secondary, confirmatory test, such as tracer urea breath testing at increased cost. Ideally, a better monoclonal stool immunoassay is needed to improve utility. Here, we examine the performance of the LIAISON® Meridian *H. pylori* SA test for the primary diagnosis as well as in patients returning for a second biopsy following treatment. This new test is a fully automated chemiluminescent immunoassay that detects the presence of *H. pylori* antigen in human stool using unique monoclonal antibodies.

## 2. Materials and Methods

### 2.1. Subjects

Subjects were enrolled from 11 sites in the U.S. and 1 site in Europe between February 2017 and August 2018. The study population was comprised of consenting adults (≥22 years) of either gender, undergoing an esophagogastroduodenoscopy (EGD) and gastric biopsy to determine *H. pylori* infection status pretherapy. Subjects that had ingested compounds that may interfere with the detection of *H. pylori* (PPI, 4 weeks of antibiotics or 2 weeks of bismuth preparations) were excluded from the study. The patients were instructed to collect a stool sample within 7 days of biopsy. Stool samples were frozen upon collection at each enrollment site and shipped to the testing sites. Posttreatment gastric biopsy and stool were analyzed in 8 noneradicated patients and tested to confirm persistent *H. pylori* infection. The study was approved by the respective local IRB committees. The clinical trial identifiers are NCT03060746 (pretherapy) and NCT03060733 (posttherapy).

### 2.2. Biopsy Testing

Specimens of the stomach were tested by at least two of three methods, known as the Composite Reference Method (CRM, considered the standard for diagnosing *H. pylori* infections): (1) histological evaluation, (2) culture of the organism, and (3) rapid urease test (RUT). A minimum of 2 positives of the 3 CRM methods was required for the diagnosis of true infection.

### 2.3. Stool Testing

*H. pylori* was detected using a new automated LIAISON® Meridian *H. pylori* SA assay (DiaSorin, Stillwater, MN). The test is a chemiluminescent immunoassay (CLIA) in sandwich format that uses novel monoclonal antibodies for capture and detection of the *H. pylori* stool antigen. Testing was performed following the manufacturer's instructions at 3 testing sites (DiaSorin (Stillwater, MN), ARUP (Salt Lake City, UT), and Northwell Health (New York, NY)). Specimens were classified as negative, equivocal, or positive based on their index (<0.9, 0.9-1.1, and >1.1, respectively).

Reproducibility testing was assessed across three investigational sites including one internal site. Six clinically negative stool matrix samples that were spiked with recombinant *H. pylori* antigen at three different concentrations (high negative, low positive, and moderate positive) were tested in duplicate using two positive and negative kit controls. Concentrations of antigen in stool were assayed in replicates of 3, in 2 runs per day over 5 operating days with 2 technicians at each site performing the test every day. A total of 90 observations occurred for each panel member. Mean, standard deviation, and coefficient of variation (%CV) were calculated using within-run, within-day, site-to-site, and total variability parameters.

### 2.4. Statistical Analysis

MedCalc 18.11.6 was utilized for all analyses presented.

## 3. Results

**Figure 1:**
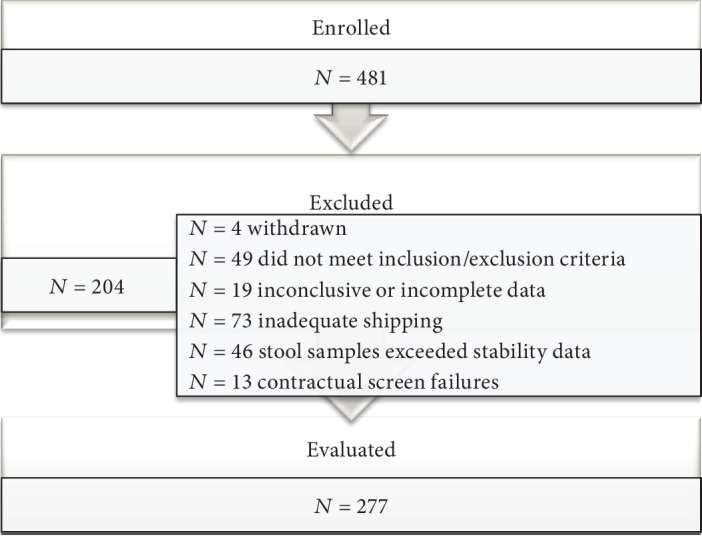
Flow diagram of the enrolled patients.

**Figure 2:**
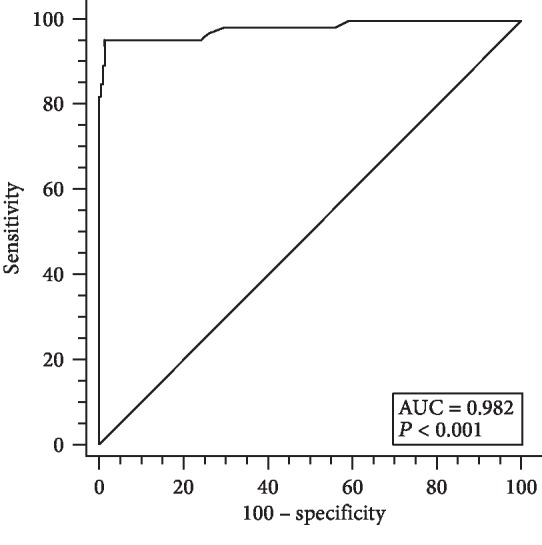
Receiver operating curve for the diagnosis of *H. pylori* infection using the LIAISON® Meridian *H. pylori* SA assay test in a group of 277 subjects undergoing esophagogastroduodenoscopy. Diagnosis was established by the Composite Reference Method. Area under the curve (AUC) = 0.982 (0.959-0.994 95% CI). Youden Index = 0.941 (0.861-0.980 95% CI).

Clinical assessment of the new LIAISON® Meridian *H. pylori* SA stool assay was performed in a multicenter clinical trial. In total, 481 patients were screened and 204 were excluded due to treatment with PPIs or antibiotics or discordant CRM outcomes (positive and negative results for 2 of the CRM methods and not tested on the third method) or incomplete data due to protocol deviation at the recruitment sites (data available for only one of the CRM methods), leaving 277 patients for enrollment (Figure 1). The basic characteristics of the enrolled subjects are shown in Table 1, with no significant differences between infected and noninfected subjects, except for race. The prevalence of infected subjects was found to be 24.2% in this study cohort, and 40% of the subjects were residents in the United States. A receiver operating characteristic (ROC) analysis was performed to locate a cut point suitable for dichotomizing the subjects into negative and positive for *H. pylor*i based on their CRM results (Figure 2). The Youden Index, calculated to be *J* = 0.941, was used to set the equivocal range of the LIAISON® Meridian *H. pylori* SA assay between 0.9 and 1.1. Clinical performance assessed against the CRM (minimum of 2 positives from histopathology, rapid urease test, or culture biopsy) showed very good agreement, with good sensitivity (95.5%) and specificity (97.6%) (Table 2). In Table 3, the LIAISON® Meridian *H. pylori* SA assay results are compared separately against histopathology, rapid urease test, and culture. Histopathology and rapid urease test results were in total agreement: although the culture test was not performed on all the biopsies, of those tested, only one did not agree with the histopathology and rapid urease test results (1 vs. 45).

Posttreatment samples of patients that returned for a second gastric biopsy and stool collection were tested (*N* = 8), and in all cases, the culture and histopathology remained positive, as was the result for the LIAISON® Meridian *H. pylori* SA test, indicating that these patients were resistant to antibiotic treatment. Clarithromycin resistance presents in about >15% of the patients worldwide (U.S., 10%) [25].

Reproducibility study results are shown in Table 4 with total variability ranging from 3.1% to 13.9% CV, site-to-site variability ranging from 2.2% to 12.5% CV, and within-run and within-day imprecision between 1.3% and 6.6% CV.

## 4. Discussion

Herein, we have shown that the new automated LIAISON® Meridian *H. pylori* SA assay provides reliable noninvasive testing for active *H. pylori* infection that is in remarkable agreement with the current, more invasive biopsy-based methods such as histology, culture, or urease. Consensus panels have advised that patients diagnosed with *H. pylori* infection should receive curative treatment because of the risk for associated adverse outcomes [26]. Furthermore, many *H. pylori*-infected individuals will not be diagnosed with an active infection or with a related disease unless reliable, convenient, and noninvasive tests are available. Diagnosis, preferably made at the primary care level, points to the use of fecal antigen testing because of its ability to detect minimal quantities of antigen in stool samples (~ng/mL), and the clinic approach conveniently shifts most of the onus of sample collection to the patient. As such, high-quality assays are needed to optimize clinical management.

Using a new monoclonal antibody sandwich method and chemiluminescent immunoassay technology, this study assessed 277 subjects in the United States and Europe, of which 24.2% were determined to have active *H. pylori* infection by CRM. The study showed a sensitivity of 95.5% (95% CI: 87.5-99.1%) and a specificity of 97.6% (95% CI: 94.5-99.2%), and the performance was comparable to a study performed in Europe using a previous version of the LIAISON kit that used different antibodies in the assay setup [27]. The current results also compare favorably with other widely marketed stool antigen tests. The package insert for the Premier Platinum HpSA enzyme immunoassay (which uses a mixture of monoclonal antibodies) reports a sensitivity of 96.1% and a specificity of 95.7% [28]. Similarly, another enzyme-based immunoassay (TechLab) indicates a sensitivity of 100% (95% CI: 89.3%-98.9%) and a specificity of 95.7% (95% CI: 89.2%-98.7%) with a smaller sample size (*n* = 109) [29]. As such, the new LIAISON® Meridian *H. pylori* SA assay appears to provide a robust alternative to older and still widely used tests. Comparative head-to-head testing is unlikely to be done due to logistical obstacles.

Multiple factors determine test availability including cost, clinical setting capabilities, pretest probability, and concomitant or recent use of antibiotics, PPIs, and bismuth that reduce the test accuracy due to suppression of the density of active *H. pylori* infection [30]. No current approach completely surmounts these issues. Some may argue about testing strategies and reserve endoscopy for those with alarm criteria or treatment failures [31]. The concern over false-negative test outcomes may warrant postponing sensitivity testing with endoscopy until a sufficient treatment washout time has elapsed. However, a better test, such as a highly sensitive fecal antigen test, may, in part, overcome these concerns. For patients unable to temporarily stop using PPIs, a positive *H. pylori* stool antigen test result represents a true-positive outcome, whereas a negative test result may represent a false-negative outcome. The latter scenario should evoke repeat testing two weeks after stopping PPI and/or antibiotic therapy. Polyclonal stool antigen tests are considered less accurate than monoclonal antibodies when compared using immunoassays (ELISA or CLIA) [32]; as such, there is consensus to avoid polyclonal stool antigen testing [33–35].

*H. pylori* antigens may be shed for a considerable time after treatment, and as such, it has been recommended that eradication testing be postponed until 6 weeks after the end of therapy because a negative test outcome may represent a false negative. Positive *H. pylori* antigen test results can be considered true-positive outcomes, but early negative testing should be confirmed with repeat testing once factors that could contribute to false-negative outcomes are mitigated. For patients with severe dyspeptic symptoms, antacids or histamine-2 receptor antagonists are a reasonable alternative to PPI therapy that does not interfere with testing [36].

We conclude that the automated LIAISON® Meridian *H. pylori* SA assay brings reliable noninvasive testing for *H. pylori* to the laboratory that is in very good agreement with the current, more invasive biopsy-based methods such as histology, culture, or rapid urease test.

## Figures and Tables

**Table 1:** Basic characteristics of the subjects according to infection status.

	Infected	Not infected	*P* value
Sex			0.204
Male	25 (37%)	61 (29%)	
Female	42 (63%)	149 (71%)	
Age			0.118
22-35	11 (16%)	34 (16%)	
36-45	16 (24%)	39 (19%)	
46-55	16 (24%)	44 (21%)	
56-65	15 (22%)	32 (15%)	
>65	9 (13%)	61 (29%)	
Race			0.0001
White	50 (75%)	192 (91%)	
Nonwhite	17 (25%)	18 (9%)	
Origin			0.021
U.S.	19 (28%)	93 (44%)	
Europe	48 (72%)	117 (56%)	

**Table 2:** LIAISON® Meridian *H. pylori* SA assay clinical performance in relation to the Composite Reference Method (CRM).

	LIAISON® Meridian *H. pylori* SA	
		95% CI
Sensitivity	95.5%	87.5-99.1
Specificity	97.6%	94.5-99.2
Positive predictive value	92.8%	84.3-96.8
Negative predictive value	98.6%	95.8-99.5
Interrater agreement (kappa)	0.922	0.870-0.975

**Table 3:** Comparison of results obtained by biopsy methods and the LIAISON® Meridian *H. pylori* SA assay for the assessment of *H. pylori* infection.

		LIAISON® Meridian *H. pylori* SA		
		Positive	Negative	Equivocal
Histopathology				
Positive	67 (24.2%)	64 (23.1%)	3 (1.1%)	0
Negative	210 (75.8%)	3 (1.1%)	205 (74.0%)	2 (0.7%)
Culture				
Positive	46 (16.6%)	45 (16.2%)	1	0
Negative	118 (42.6%)	3 (1.1%)	113 (40.8%)	2 (0.7%)
Not performed	113 (40.8%)	19 (6.9%)	94 (33.9%)	0
Rapid urease test				
Positive	67 (24.2%)	64 (23.1%)	3 (1.1%)	0
Negative	210 (75.8%)	3 (1.1%)	205 (74.0%)	2 (0.7%)

**Table 4:** Reproducibility of the LIAISON® Meridian *H. pylori* SA test assessed over 5 days at multiple sites.

	Mean index value	Within run		Day to day within site		Site to site		Total	
		SD	%CV	SD	%CV	SD	%CV	SD	%CV
Neg Ctrl	0.07	0.004	5.10%	0.002	2.10%	0.009	12.50%	0.010	13.90%
Pos Ctrl	4.80	0.076	1.60%	0.063	1.30%	0.105	2.20%	0.153	3.10%
Mod Pos sample #1	2.12	0.034	1.60%	0.108	5.10%	0.119	5.60%	0.168	8.00%
Mod Pos sample #2	2.37	0.049	2.10%	0.156	6.60%	0.226	9.50%	0.283	11.90%
High Neg sample #1	0.69	0.024	3.50%	0.037	5.40%	0.065	9.40%	0.081	11.80%
High Neg sample #2	0.69	0.023	3.30%	0.019	2.70%	0.065	9.40%	0.077	11.00%
Low Pos sample #1	1.21	0.031	2.50%	0.029	2.40%	0.093	7.70%	0.109	9.00%
Low Pos sample #2	1.20	0.021	1.70%	0.056	4.70%	0.120	10.10%	0.138	11.50%

## Data Availability

Raw data will be available upon request.

## Abbreviations

PPI: Proton pump inhibitor
SAT: Stool antigen test
ROC: Receiver operating characteristic
CRM: Composite Reference Method
AUC: Area under the curve
H2: Type 2 histamine receptor.
